# Cationic Glucan Dendrimer Gel-Mediated Local Delivery of Anti-OC-STAMP-siRNA for Treatment of Pathogenic Bone Resorption

**DOI:** 10.3390/gels10060377

**Published:** 2024-05-31

**Authors:** Kenta Yamamoto, Shin-Ichi Sawada, Satoru Shindo, Shin Nakamura, Young M. Kwon, Nazanin Kianinejad, Saynur Vardar, Maria Hernandez, Kazunari Akiyoshi, Toshihisa Kawai

**Affiliations:** 1 Department of Immunology, Kyoto Prefectural University of Medicine, Kyoto 602-8566, Japan; 2 Department of Oral Science and Translational Research, College of Dental Medicine, Nova Southeastern University, Fort Lauderdale, FL 33328, USA; sshindo1@nova.edu (S.S.); snakamur@nova.edu (S.N.); tkawai@nova.edu (T.K.); 3 Department of Polymer Chemistry, Graduate School of Engineering, Kyoto University, Kyoto 605-0981, Japan; shsawada@chiba-u.jp (S.-I.S.); akiyoshi@bio.polym.kyoto-u.ac.jp (K.A.); 4 Synergy Institute for Futuristic Mucosal Vaccine Research and Development, Chiba University, Chiba 260-8670, Japan; 5 Department of Pharmaceutical Sciences, College of Pharmacy, Nova Southeastern University, Fort Lauderdale, FL 33328, USAnk454@mynsu.nova.edu (N.K.); 6 Department of Periodontology, College of Dental Medicine, Nova Southeastern University, Fort Lauderdale, FL 33328, USA; svardarsengul@nova.edu (S.V.); marher@nova.edu (M.H.)

**Keywords:** cationic glycan dendrimer, drug delivery systems, regenerative medicine, OC-STAMP, siRNA

## Abstract

Osteoclast stimulatory transmembrane protein (OC-STAMP) plays a pivotal role in the promotion of cell fusion during osteoclast differentiation (osteoclastogenesis) in the context of pathogenic bone resorption. Thus, it is plausible that the suppression of OC-STAMP through a bioengineering approach could lead to the development of an effective treatment for inflammatory bone resorptive diseases with minimum side effects. Here, we synthesized two types of spermine-bearing (Spe) cationic glucan dendrimer (GD) gels (with or without C12) as carriers of short interfering RNA (siRNA) to silence OC-STAMP. The results showed that amphiphilic C12-GD-Spe gel was more efficient in silencing OC-STAMP than GD-Spe gel and that the mixture of anti-OC-STAMP siRNA/C12-GD-Spe significantly downregulated RANKL-induced osteoclastogenesis. Also, local injection of anti-OC-STAMP-siRNA/C12-GD-Spe could attenuate bone resorption induced in a mouse model of periodontitis. These results suggest that OC-STAMP is a promising target for the development of a novel bone regenerative therapy and that C12-GD-Spe gel provides a new nanocarrier platform of gene therapies for osteolytic disease.

## 1. Introduction

Small interfering RNA (siRNA) is a nucleic acid fragment containing 21–25 nucleotides that could specifically suppress the expression of a target gene. Accordingly, the use of RNA interference (RNAi) molecules offers a novel approach to tissue regeneration [1,2]. The specificity of inhibition is driven by complementary base pairing between siRNA and target messenger RNA (mRNA), which leads to the recruitment of RNA-induced silencing complex (RISC) proteins that cleave target mRNA [3]. However, in general, siRNAs can be easily degraded by RNase, resulting in poor permeation of the cell surface membrane owing to high molecular weight (MW) and negative charges. This means that the delivery of naked siRNAs is less than ideal. To address this obstacle, several nanocarriers, such as liposomes, dendrimers, and block copolymers, have been developed for siRNA delivery into target cells with some promising results [4,5,6].

**Figure 1:**
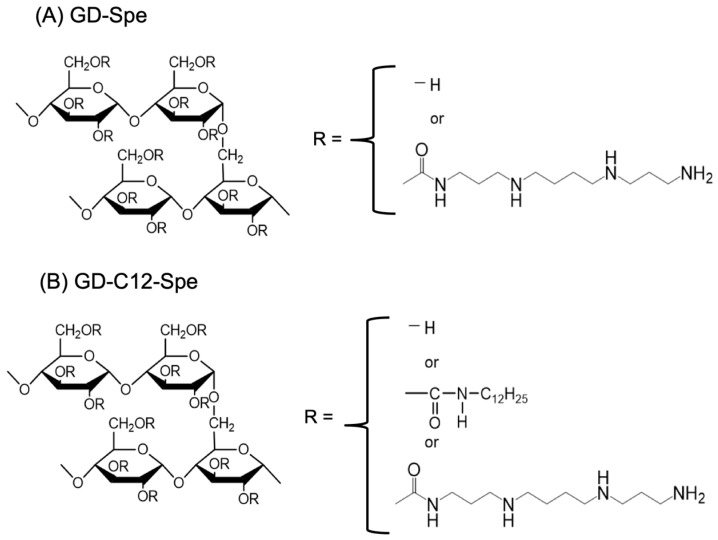
Chemical structure of GD-Spe and C12-GD-Spe. (**A**) GD-Spe: Chemical structure of spermine (Spe)-bearing glucan dendrimer (GD). R can be replaced with H or spermine (approximately 10:1 ratio). (**B**) C12-GD-Spe: Chemical structure of C12- and Spe-bearing glucan dendrimer (GD). R can be replaced with H, spermine, or C_12_H_25_-NHCO-(approximately 10:1:0.1 ratio).

Here, we synthesized two types of spermine-bearing glucan dendrimer (GD) gels as new nanocarriers for siRNA delivery: (1) GD-Spe and (2) dodecyl isocyanate (C12)-GD-Spe amphiphilic (Figure 1). The main body of these cationic GD gels is composed of naturally occurring polysaccharides and spermine, both of which are bioabsorbable with no detrimental impact on recipient cells and tissue. Spermine, an endogenously available polyamine that possesses a strong positive charge, can tightly bind negatively charged nucleic acids, such as DNAs and RNAs. It is also true that spermine can condense DNA and RNA in sperm owing to its strong positive charge in the physiological condition [7,8]. Therefore, spermine-bearing GD gel is expected to hold its molecular cargos, i.e., siRNA, tightly. Also, since the total charge of the spermine-bearing-GD/siRNA complex remains positive, it will adhere to the negatively charged cell membrane surface, thereby allowing the sufficient and efficient cellular uptake of siRNA. In addition, we have previously reported a spermine-modified amylose-based nanocarrier gel that could effectively deliver CpG DNA and siRNA [9,10].

Constant bone mass is maintained by homeostatic bone remodeling consisting of coordinated sequential actions of osteoclast-mediated bone resorption followed by osteoblast-mediated bone formation, i.e., osteoblast-osteoclast coupling [11,12]. In the context of homeostatic bone remodeling, osteoclasts act on osteoblast lineage cells by providing osteoblastogenesis factors, such as IGF-1 and TGF-β, which are released from resorbed bone matrix or bone-resorbing osteoclasts by themselves [13]. Therefore, the coupling of bone formation to resorption plays a pivotal role in healthy bone remodeling. However, in some bone lytic conditions, such as osteoporosis and periodontitis, homeostatic bone remodeling is disrupted [14,15]. Pharmacological approaches for bone lytic disease are generally divided into two types [16,17]. One involves the administration of an anabolic agent, such as parathyroid hormone or monoclonal antibody for sclerostin, to activate osteoblasts. The other involves the administration of anti-resorptive agents targeting osteoclasts, such as bisphosphonates or monoclonal antibodies for RANKL. Although bisphosphonates, as anti-resorptive agents, are the most commonly and frequently used osteoporosis treatment for their high effectiveness, they sometimes induce severe side effects, including medication-related osteonecrosis of the jaws (MRONJ) and atypical femoral fractures, owing to reduction of homeostatic bone resorption [18,19,20]. Thus, targeting molecules only associated with pathological bone resorption is more desirable.

It has been reported that two fusogens, osteoclast stimulatory transmembrane protein (OC-STAMP) and dendritic cell–specific transmembrane protein (DC-STAMP), are required to form multinuclear (mature) osteoclasts from their mononuclear precursors in response to stimulation with osteoclastogenesis factor, i.e., receptor activator of nuclear factor kappa-B ligand (RANKL) [21,22]. However, among monocyte/macrophage lineage cells, the respective expression of these two fusogens is limited in their mononuclear precursors [23]. Unlike the G protein-coupled receptor (GPCR) family of proteins with seven transmembrane-spanning domains, the results of in silico structure prediction show that both STAMP molecules in mice are composed of six transmembrane-spanning domains and three extracellular loops [22]. Therefore, OC-STAMP is structurally similar to DC-STAMP and contains a region with relatively high homology to DC-STAMP. Nonetheless, recent studies have revealed differences between them. Specifically, DC-STAMP-KO mice showed apparent osteopetrotic phenotypes, while in OC-STAMP-KO mice, little or no bone skeletal phenotypes have been observed [24,25]. Therefore, the literature seems to have settled on a consensus that holds that OC-STAMP is not associated with any pathogenic bone disorders. Nonetheless, OC-STAMP-KO mice did show an apparent lower alveolar bone loss in a periodontal disease model, indicating that OC-STAMP is mainly engaged in pathogenic bone resorption in periodontitis but not in homeostatic bone remodeling. This finding stands in contrast to that of DC-STAMP, which is engaged in both homeostatic and pathogenic bone remodeling [26]. These findings lead to the hypothesis that inhibition of OC-STAMP expression at the local bone resorption site of periodontitis would lead to the development of a novel therapeutic regimen.

Accordingly, we herein asked if cationic GD gel would work as a drug delivery system (DDS) for xOC-STAMP-siRNA and followed up by investigating the possible efficacy of xOC-STAMP-siRNA/cationic GD complex in treating pathogenic bone resorption induced in the mouse model of ligature-induced periodontitis.

## 2. Results

### 2.1. Characteristics of Cationic Glucan Dendrimer

**Figure 2:**
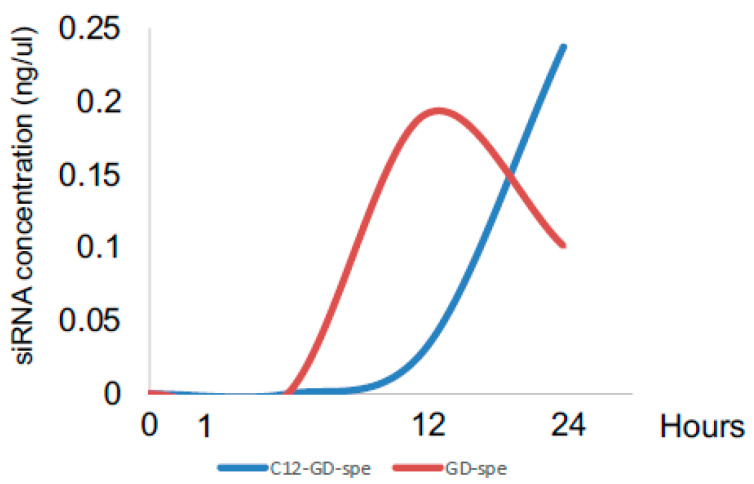
Release of siRNA from GD-Spe and C12-GD-Spe gels. The amount of siRNA released from each type of gel at 12 and 24 h was monitored. Results demonstrated that C12-GD-Spe gels can provide more extended release of siRNA than GD-Spe gels.

According to dynamic light scattering and zeta potential analyses, GD-Spe and C12-GD-Spe gel were found to be positively charged monodisperse nanoparticles about 20 nm in diameter (GD-spe: diameter 19.2 ± 0.9 nm and zeta potential +9.10; and C12-GD-spe: diameter 22.8 ± 0.8 and zeta potential +4.19). After the incubation of respective siRNA-loaded gels in PBS, the peak release of siRNA from GD-Spe and C12-GD-Spe gels was detected at 12 and 24 h, respectively (Figure 2). In sum, the results demonstrated that C12-GD-Spe gels can facilitate a more extended release of siRNA than GD-Spe gels, indicating the superior efficiency of siRNA delivery by C12-GD-Spe gels compared to that of GD-Spe gels.

### 2.2. Evaluation of Cationic Glucan Dendrimer as a Candidate Nanocarrier for RNA Silencing

**Figure 3:**
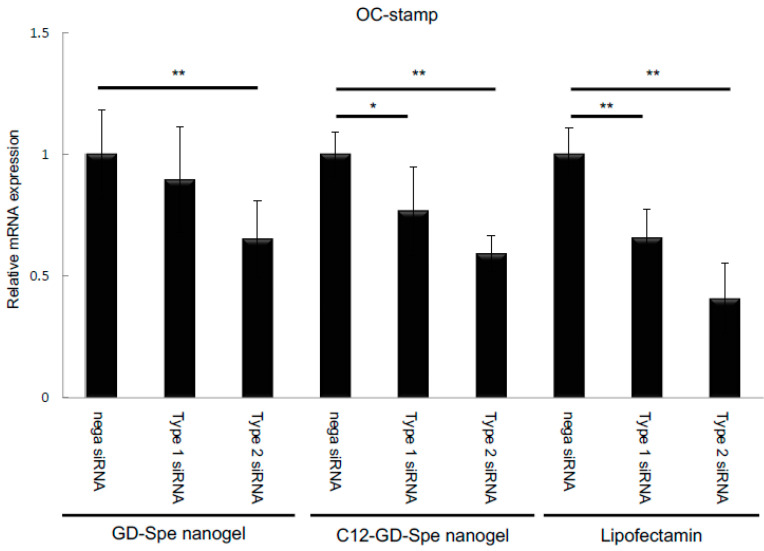
Comparison of OC-STAMP gene inhibition of xOC-STAMP-siRNA1 and -siRNA 2 on RAW264.7 cells. RAW264.7 cells were treated with xOC-STAMP-siRNA1 or -siRNA2 mixed with GD-Spe gel, GD-C12-Spe gel, or Lipofectamine and stimulated with RANKL. Results demonstrated that xOC-STAMP-siRNA2 suppressed OC-STAMP mRNA expression more than xOC-STAMP-siRNA1 for each reagent on RAW264.7 cells. * *p* < 0.05% and ** *p* < 0.01% vs. each group.

We first evaluated the capacity of cationic GD gel to serve as a nanocarrier by using two types of siRNA (xOC-STAMP-siRNA-1 and xOC-STAMP-siRNA-2, as described in the Section 4) for silencing OC-STAMP mRNA expression in RANKL-stimulated RAW264.7 cells. The two types of siRNA were mixed with GD-Spe, GD-C12-Spe, or Lipofectamine (positive control), respectively, and transfected to RAW264.7 cells. Cells were then stimulated with RANKL for 2 days. OC-STAMP expression was significantly downregulated in xOC-STAMP-siRNA-2/GD-Spe, xOC-STAMP-siRNA-1/C12-GD-Spe, and xOC-STAMP-siRNA-2/C12-GD-Spe groups, but not the xOC-STAMP-siRNA-1/GD-Spe group (Figure 3). These results showed that both GD-Spe and C12-GD-Spe gel could silence the expression of OC-STAMP mRNA in RANKL-stimulated RAW264.7 cells with results matching those of the Lipofectamine group, which was used as positive control, albeit with slightly lower efficiency. Across groups, xOC-STAMP-siRNA-2 showed lower OC-STAMP mRNA expression in all mixtures: GD-Spe (xOC-STAMP-siRNA-1: 89.5% vs. xOC-STAMP-siRNA-2: 69.2%), GD-C12-Spe (xOC-STAMP-siRNA-1: 76.6% vs. xOC-STAMP-siRNA-2: 59.1%), and Lipofectamine (xOC-STAMP-siRNA-1: 65.7% vs. xOC-STAMP-siRNA-2: 40.7%) (Figure 3). Therefore, xOC-STAMP-siRNA-2 was used as a representative siRNA in the following experiments.

### 2.3. Comparing Efficacy of the Two GDs for OC-STAMP Silencing on BMMCs

**Figure 4:**
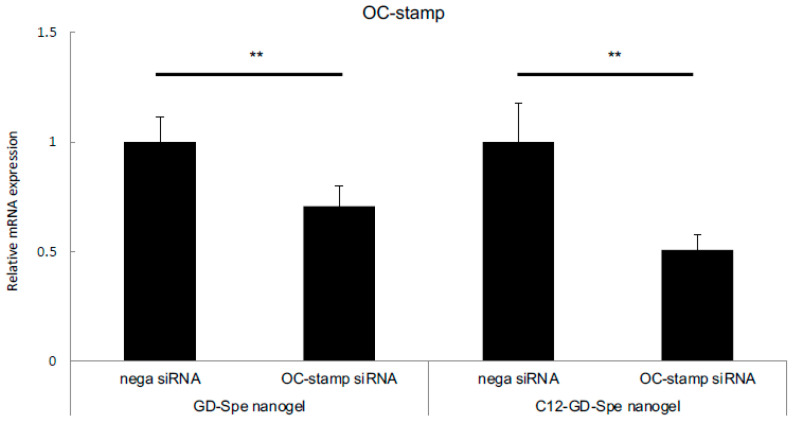
Comparison of OC-STAMP gene inhibition of GD-Spe and C12-GD- Spe gels on BMMCs. BMMC cells were treated with negative control or xOC-STAMP-siRNA mixed with GD-Spe or C12-GD-Spe gels and stimulated with M-CFS and RANKL. Again, C12-GD-Spe gel suppressed OC-STAMP mRNA expression more than GD-Spe gel on BMMCs. ** *p* < 0.01% vs. each group.

Next, we compared the two types of GD gels as siRNA nanocarriers for their silencing efficiency in OC-STAMP expression using primary BMMCs. Both GD gels showed significant silencing of OC-STAMP expression on RANKL-stimulated BMMCs, but xOC-STAMP-siRNA/C12-GD-Spe downregulated OC-STAMP expression more effectively compared to xOC-STAMP-siRNA/GD-Spe (70.7% vs. 50.8%) (Figure 4), and this result is comparable to that of experiments using RAW264.7 cells (Figure 2). Thus, we used the C12-GD-Spe for further experiments. 

### 2.4. Effects of xOC-STAMP-siRNA/C12-GD-Spe Complex on Osteoclastogenesis of BMMCs

**Figure 5:**
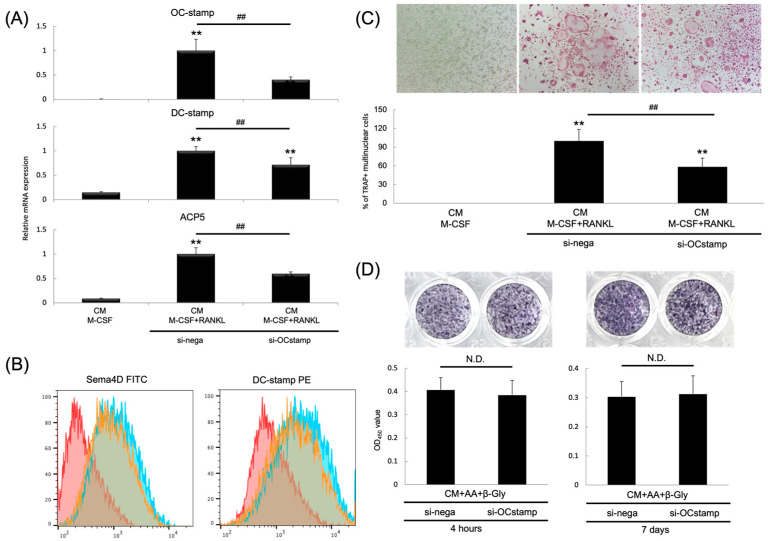
Effects of xOC-STAMP-siRNA/C12-GD-Spe on the osteoclasto-genesis from RANKL-stimulated BMMCs. BMMCs were treated with negative or xOC-STAMP-siRNA mixed with C12-GD-Spe gel and cultured with M-CSF alone or M-CSF and RANKL. (**A**) xOC-STAMP-siRNA/C12-GD-Spe complex significantly suppressed OC-STAMP, DC-STAMP, and ACP5 mRNA expression in BMMCs stimulated with RANKL. (**B**) xOC-STAMP siRNA/C12-GD-Spe also downregulated DC-STAMP and Sema4D cell surface protein expression. Blue histogram: negative control siRNA/C12-GD-Spe and RANKL applied to BMMCs; orange histogram: xOC-STAMP-siRNA/C12-GD-Spe and RANKL applied to BMMCs; red histogram: BMMCs cultured without RANKL stimulation. (**C**) xOC-STAMP-siRNA/C12-GD-Spe significantly inhibited the formation of TRAP-positive multinuclear cells. ** *p* < 0.01% vs. M-CSF alone. ## *p* < 0.01% vs. each group. (**D**) MC3T3-E1 were treated with negative or xOC-STAMP-siRNA mixed with C12-GD-Spe gel for 4 h or 7 days, and then ALP staining was performed. xOC-STAMP-siRNA/C12-GD-Spe did not affect ALP activity of the osteogenic cells. N.D. = no difference.

We next evaluated if silencing OC-STAMP induced by xOC-STAMP-siRNA/C12-GD-Spe could downregulate osteoclastogenesis (OC-genesis) of BMMCs stimulated by RANKL. We found that the mRNA expression level of ACP5 (Acid Phosphatase 5, Tartrate Resistant) and DC-STAMP, which are osteoclast markers, was significantly downregulated in the xOC-STAMP-siRNA/C12-GD-Spe group (ACP5: 59.6%, DC-STAMP: 71.4%) (Figure 5A). Downregulation of OC-STAMP mRNA expression (40.3%) in the experiments was also confirmed (Figure 5A). Se-ma4D, previously known as an axon guidance molecule, is derived from osteoclasts and plays an anti-coupling role by suppressing osteoblastogenesis. Interestingly, both Sema4D and DC-STAMP cell surface protein expressions were attenuated in the xOC-STAMP-siRNA/C12-GD-Spe group (Figure 5B), and the complex significantly reduced the number of TRAP-positive multinuclear cells (58.5%) (Figure 5C). xOC-STAMP-siRNA/C12-GD-Spe complex did not upregulate ALP activity in MC3T3-E1 osteoblast precursor cells that were primed with β-glycerophosphate and ascorbic acid (Figure 5D). However, Sema4 secretion from OCs was attenuated by the xOC-STAMP-siRNA/C12-GD-Spe complex, suggesting that xOC-STAMP-siRNA/C12-GD-Spe can promote the osteoblastogenesis via downregulating the production of Sema4D, an anti-coupling factor, by osteoclasts. Meanwhile, it is clear that xOC-STAMP-siRNA/C12-GD-Spe complex can downregulate RANKL-induced osteoclastogenesis in BMMCs in vitro. 

### 2.5. Effects of OC-STAMP siRNA/C12-GD-Spe Complex on Alveolar Bone Resorption in Ligature-Induced Periodontitis

**Figure 6:**
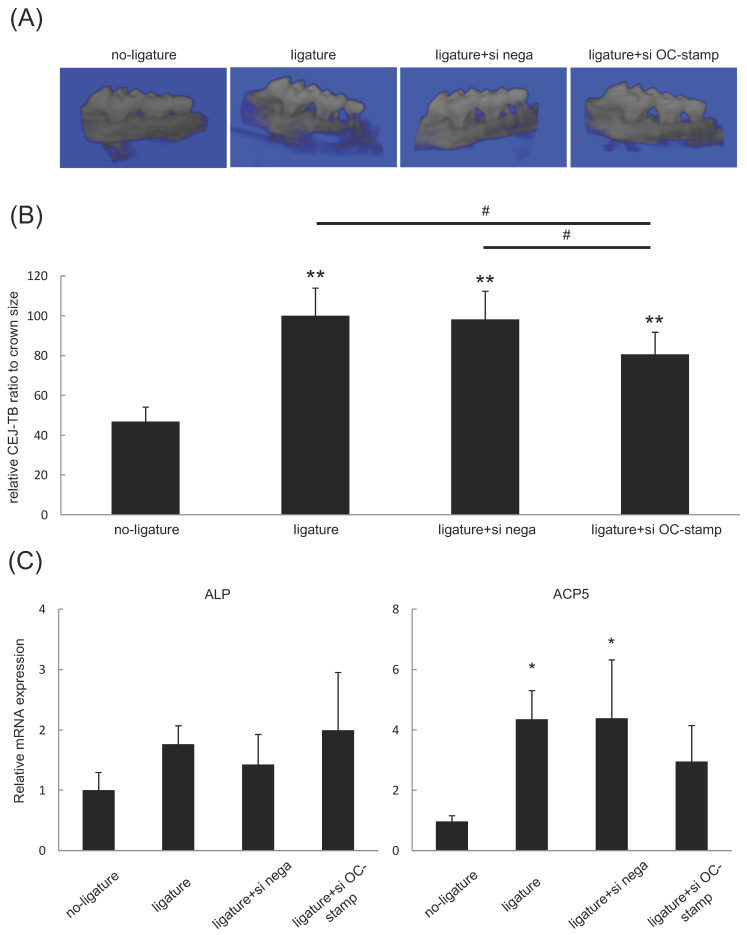
Effects of xOC-STAMP-siRNA/C12-GD-Spe on bone resorption in ligature-induced mouse periodontitis model. Periodontitis was induced by the placement of silk ligature around the cervix of maxillary second molar. After 1 week of ligature placement, negative control siRNA or xOC-STAMP-siRNA/C12-GD-Spe was injected into the gingival tissue adjacent to the ligatured site. Then, alveolar bone resorption was evaluated by μCT imaging. (**A**) Micro CT imaging of each group. (**B**) xOC-STAMP-siRNA/C12-GD-Spe-injected mice showed less alveolar bone loss than ligatured mice. (**C**) Local injection of xOC-STAMP-siRNA/C12-GD-Spe did not show significant change of ALP mRNA expression compared to the groups (1) ligature alone, (2) ligature + xOC-STAMP-siRNA/C12-GD-Spe, and (3) ligature + control-siRNA/C12-GD-Spe. However, expression of ACP5 mRNA was significantly elevated in the groups of (1) ligature alone and (3) ligature + control-siRNA/C12-GD-Spe, but not in the group (2) ligature + xOC-STAMP-siRNA/C12-GD-Spe, compared to the baseline control group (no ligature). * *p* < 0.05% and ** *p* < 0.01% vs. no ligature. # *p* < 0.05% vs. each group.

We further evaluated whether the administration of OC-STAMP siR-NA/C12-GD-Spe complex could alleviate alveolar bone resorption in a ligature-induced periodontitis mouse model by μCT examination. We found that the group treated with OC-STAMP siRNA/C12-GD-Spe showed a significant reduction of relative CEJ-ABC distance ratio adjusted to tooth crown size when it was compared with the ligatured group, even though the value was still higher than that of the non-ligature group (Figure 6A,B). However, such a significant reduction was not observed in the group that received negative control siRNA/C12-GD-Spe treatment. We monitored mRNA expressions for ALP and ACP5 in the periodontal tissues isolated from the mice sacrificed on Day 7 (Figure 6C). Importantly, the levels of ALP mRNA expression were comparable in all groups, indicating that siRNA/C12-GD-Spe has little or no effect on osteoblastogenesis. On the other hand, ACP5 expression was significantly upregulated in the groups that received ligature alone (periodontitis) or ligature and control-siRNA/C12-GD-Spe treatment (periodontitis + placebo injection) compared to the baseline control group (no periodontitis). However, there was no significant difference between the group that received both ligature and OC-stamp-siRNA/C12-GD-Spe treatment (periodontitis + OC-stamp siRNA injection) and the baseline control group (no periodontitis), suggesting that OC-stamp-siRNA/C12-GD-Spe showed a trend of suppressing the bone resorption. These results indicate that C12-GD-Spe can facilitate the siRNA delivery in the in vivo context and that administration of the xOC-STAMP-siRNA/C12-GD-Spe complex could suppress alveolar bone resorption.

## 3. Discussion 

In this study, we aimed to examine the efficacy of newly designed and synthesized cationic amphiphilic GD gels as a suitable nanocarrier for siRNA delivery, both in vitro and in vivo. We also investigated the effect of OC-STAMP siRNA/GD complex on pathogenic bone loss induced in the mouse model of periodontitis. Our results demonstrated that both cationic GD-Spe-containing xOC-STAMP-siRNAs could knock down the expression of OC-STAMP mRNA, the target gene of siRNA, and that C12-GD-Spe gel, in particular, was more efficient than GD-Spe gel in delivering the gene. Moreover, the xOC-STAMP-siRNA/C12-GD-Spe complex effectively attenuated RANKL-induced osteoclastogenesis of BMMCs and inhibited alveolar bone loss caused by ligature-induced periodontitis.

Nanocarriers for drug delivery have been developed using various self-assembled systems, such as liposomes, polymeric micelles, and polymeric vesicles [27,28,29]. Such self-assembly-based systems have shown high potential as drug delivery systems (DDS), but they are generally unstable owing to their sensitivity to the local environment. On the other hand, hyperbranched polymers have better stability [30]. In addition, natural polysaccharides have been widely used as biomaterials in various nanocarriers because of their biocompatibility and biodegradability [31]. Indeed, self-assembled nanogels of hydrophobically modified polysaccharides have shown promising outcomes for siRNA delivery carriers [9]. Glycogen is representative of a naturally occurring hyperbranched polysaccharide consisting of α-(1,4),(1,6)-linked glucose units [32]. Thus, in this study, we used hyperbranched α-glucans synthesized by glycogen branching enzyme, sucrose phosphorylase, and α-glucan phosphorylase [33]. In general, the positive electrical charge of the gel surface promotes delivery efficiency to target cells by the negative charge of the cellular membrane, and a stronger positive charge correlates with stronger delivery efficiency. Even though GD-Spe gel has a slightly stronger positive charge compared to dodecyl isocyanate-modified C12-GD-Spe gel (Section 4), C12-GD-Spe gel showed higher efficiency in silencing the OC-STAMP gene than that shown by GD-Spe gel in our study. This could be attributed to the amphiphilic nature of C12-GD-Spe gel by the introduction of the hydrophobic group. The long alkyl chains attached to GD strengthened hydrophobic interactions with the biomembrane and, hence, achieved greater delivery efficiency. In the present study, we found that C12-GD-Spe gel released siRNA for a longer period than GD-Spe gel (Figure 2), illustrating the superior delivery property of C12-GD-Spe gel compared to GD-Spe gel. It is noteworthy that the above-noted findings in this study are consistent with those of a previous study that used C12-GD for protein delivery [33]. 

Periodontal disease is one of the most prevalent chronic infectious diseases in humans, affecting more than 60% of adults [34]. It is the main cause of tooth loss and a consequent decline in the quality of life [34]. Pathological features of periodontal disease are characterized by chronic inflammation of periodontal soft tissue and alveolar bone loss mediated by oral microbial dysbiosis [35]. In periodontal disease, excessive osteoclastogenesis is induced by local production of RANKL from bacteria-reactive immune cells [35]. Osteoporosis is another progressive bone disorder characterized by pathologically elevated RANKL-dependent osteoclastogenesis, which, in turn, results in low bone density, especially found in older women, and it results in significant bone fractures [16]. It is the pathological hyperactivity of osteoclasts that tips the balance between bone-forming osteoblasts and bone-resorbing osteoclasts in the context of osteoporosis [16]. The onset and progression of both bone lytic diseases are assumed to increase as the population advances in age, requiring expensive, long-term medical and dental care. Viewed from that prism, the identification of new targets against hyperactive OC-genesis is clinically urgent.

RNA silencing could have a significant impact on bone regeneration because of its remarkable capacity to alter cell behavior through highly specific gene translational silencing. Accordingly, much research has focused on several genes related to bone remodeling [36,37]. RNA silencing for bone regeneration is largely divided into two approaches. One targets negative regulatory genes for osteogenic differentiation on pre-osteoblasts and -osteoblasts. The other silences genes that facilitate osteoclastogenesis on monocytes and pre-osteoclasts. In the first, specific siRNA for Noggin [38] and Sema4D [39] was used, which resulted in enhanced bone regeneration. In the second, siRNA targeting RANK, NFATc1, and DC-STAMP, all engaged in osteoclastogenesis, has been reported to significantly inhibit osteoclastogenesis and prevent bone loss in each disease model [40,41,42]. These results support siRNA as a nanocarrier delivery system for bone treatment. However, silencing such genes may induce side effects, such as MRONJ and atypical femoral fracture, by reducing homeostatic bone resorption through the induction of RANKL antibodies or bisphosphonates. On the other hand, OC-STAMP can mediate osteoclastogenesis as a fusogen in pathological, but not physiological, conditions [24,25]. Moreover, anti-OC-STAMP antibody treatment was more effective in the prevention of alveolar bone loss (about 50% suppression) than anti-DC-STAMP antibody (about 25% suppression) in ligature-induced periodontitis [24,43]. These reports indicate that treatment using specific siRNA for OC-STAMP has potential, especially in pathological bone lytic conditions like severe periodontal disease and osteoporosis. In our study, we found that the xOC-STAMP-siRNA/C12-GD-Spe complex could downregulate osteoclastogenesis and ameliorate bone resorption, similar to findings in other reports, using siRNA-targeting genes involved in osteoclast development. To the best of our knowledge, this is the first report to assess the effect of nanocarrier-based xOC-STAMP-siRNA delivery to silence OC-STAMP mRNA expression in osteoclasts. 

However, it should be noted that this study is limited because we only showed the prevention potency of bone loss induced by an acute inflammation model. Typically, bone loss caused by periodontitis and osteoporosis is the result of chronic complications, thus presenting the successful utilization of RNA-based therapy. We have only discussed bone regeneration based on the use of RNA silencing, but transplantation of osteoblasts into bone lesions can also accelerate bone healing and boost functional recovery [44]. Recently, it has been reported that functional osteoblasts can be easily obtained using a direct reprogramming technique by the transduction of some transcription factors, such as Runx2, Osterix, Oct4, and L-myc, or small-molecule compounds, such as Repsox [45,46]. Therefore, it is anticipated that combining the local delivery of siRNA targeting OC-STAMP mRNA with transplantation of directly reprogramed osteoblastic cells could result in a pharmacological synergism to facilitate the suppression of osteoclastogenesis followed by bone regeneration in the bone defects caused by bone lytic diseases. 

Taken together, the results of the present study revealed that C12-GD-Spe gel could efficiently deliver siRNA and regulate osteoclastogenesis by targeting OC-STAMP. Also, alveolar bone loss was alleviated by local administration of the xOC-STAMP-siRNA/C12-GD-Spe complex. These results suggest that OC-STAMP is a promising candidate target for the development of a novel bone regenerative therapy and that C12-GD-Spe, in particular, offers a new nanocarrier platform for gene therapies that treat osteolytic diseases.

## 4. Materials and Methods

### 4.1. Synthesis of GD-Spe and C12-GD-Spe

GD (Mw = 2.2 × 10^5^) (a gift from Ezaki Glico Co., Ltd., Osaka, Japan) was reacted with dodecyl isocyanate to obtain dodecyl-bearing GD (C12-GD). The substitution degree of dodecyl group was 3.2 in 100 glucose units of GD. Cationic spermine groups were introduced to GD and C12-GD by the conventional 1,1′-carbonyldiimidazole (CDI) activation method. The degree of substitution of spermine for GD was 25 in 100 glucose units of GD (GD-Spe), and that of C12-GD was 22 in 100 glucose units of GD (C12-GD-Spe).

### 4.2. siRNA Release from GD-Spe and C12-GD-Spe

The siRNA and GD mixture was prepared by mixing the following reagents: 1 μL of siRNA (10 μM), 2.95 μL of GD-Spe solution (1 mg/mL), and 6.05 μL of distilled water or 3.35 μL of C12-GD-Spe solution (1 mg/mL) and 5.65 μL of distilled water. After incubation for 30 min at room temperature, mixtures were centrifuged at 14,000× *g* for 10 min to remove non-reacted siRNA. The pellet of siRNA/gel mixture was resuspended in fresh water and incubated for 12 and 24 h. At each timepoint of 0, 12, and 24 h, the supernatant collected via centrifugation was subject to the measurement of siRNA concentration using Nanodrop (OD260, Thermo Fisher Scientific).

### 4.3. Cell Culture

RAW264.7 (ATCC Manassas, VA, USA) murine monocytes were maintained in complete medium (CM: α-MEM [Thermo Fisher Science, Waltham, MA, USA]) supplemented with 10% FBS (Atlanta Biologicals, Lawrenceville, GA, USA), 1% non-essential amino acid, and 1% antibiotics). Bone marrow-derived mononuclear cells (BMMCs) were established as previously described [47]. In brief, bone marrow cells isolated from the femur and tibia of C57BL/6J mice by the density gradient centrifugation method, using Histopaque 1083 (Sigma-Aldrich, St. Louis, MO, USA), were seeded on a cell culture plate and expanded in CM supplemented with 20 ng/mL M-CSF (R&D Systems, Minneapolis, MN, USA) for 3 days. The cell culture plate was washed with complete medium, and the remaining adherent cells in the plate were used as BMMCs.

### 4.4. siRNA, siRNA/GD-Spe, and siRNA/C12-GD-Spe 

Two types of siRNA targeting murine OC-STAMP, including xOC-STAMP-siRNA-1 (#SI00835926, target sequence: CCCAATCACACTCACAGTCAA) and xOC-STAMP-siRNA-2 (#SI00835940, target sequence: GAGCTTCAGTTTCCAAATCTA), were purchased from QIAGEN (Venlo, The Netherlands). Type 2 siRNA showed the higher silencing efficiency of the two (Supplementary Table S1) and was therefore used as the representative siRNA targeting murine OC-STAMP in this study. Non-silencing control siRNA (AllStars Negative Control siRNA, #1027280, QIAGEN) was used as negative control. To form siR-NA/GD-Spe and siRNA/C12-GD-Spe, 1 μL of each siRNA (10 μM) was rapidly mixed with 2.95 μL of GD-Spe solution (1 mg/mL) plus 6.05 μL of distilled water or 3.35 μL of C12-GD-Spe solution (1 mg/mL) plus 5.65 μL of distilled water, respectively, and incubated for 30 min at 25 °C so that the cation/phosphate (C/P) ratio of each resultant mixture equaled 8 (C/P = 8). Then, the respective mixture was diluted to 1:49 with Opti-MEM (Thermo Fisher Scientific) before the transfection. 

### 4.5. siRNA Transfection In Vitro 

RAW264.7 cells were seeded in wells of a 24-well cell culture plate at a density of 2 × 104 cells/well. On the next day, the above-noted working concentration of siRNA/GD-Spe (500 μL), siRNA/C12-GD-Spe (500 μL), or siRNA/Lipofectamine (Thermo Fisher Science, Waltham, MA, USA, diluted in Opti-MEM [20 nM siRNA], 500 μL) was added to wells of a culture plate and reacted for 4 h. After removing the mixtures, RAW264.7 cells were cultured in CM supplemented with 10 ng/mL RANKL for different periods of time, depending on the assays performed, including qRT-PCR, FACS and TRAP staining. BMMCs were seeded in wells of 24- and 12-well culture plates at a density of 1 × 105 and 4 × 105 cells/well, respectively, and cultured with CM supplemented with 50 ng/mL M-CSF for 4 days. For the assay using BMMCs as osteoclast precursors, BMMCs were exposed to 500 and 1000 μL of siRNA/GD-Spe or C12-GD-Spe mixture diluted with Opti-MEM (20 nM siRNA) for 4 h. Then, the cells were cultured with CM supplemented with M-CSF (50 ng/mL) with/without RANKL (10 ng/mL) for different periods of time, depending on post-culture analyses. 

### 4.6. Quantitative Real-Time Reverse Transcriptase Polymerase Chain Reaction (RT-PCR)

Forty-eight hours after siRNA treatment, RNA was isolated, and cDNA was synthesized according to a method previously described [48]. Briefly, total RNA was gained using the RNeasy mini kit (QIAGEN, Venlo, The Netherlands), and cDNA was reverse transcribed using iScript Reverse Transcription Supermix (Bio-Rad, Hercules, CA, USA). The cDNA was subjected to Real-time RT-PCR on a Step One Plus Real-Time PCR System (Applied Biosystems) with TaqMan Fast Advanced Master Mix (Applied Biosystems, Waltham, MA, USA) and matching probes and primers (Thermo Fisher Scientific) (Supplementary Table S1). All values (average ± standard deviation [SD]) were normalized to the expression level of housekeeping gene, GAPDH mRNA, in each sample and expressed as relative values. 

### 4.7. In Vitro Osteoclastogenesis Assay

The levels of osteoclastogenesis were analyzed by their expression of tartrate-resistant acid phosphate (TRAP). More specifically, using the Leukocyte Acid Phosphatase staining kit (Sigma-Aldrich, St. Louis, MO, USA), mature multinucleated osteoclasts at 7 days after siRNA treatment were identified. TRAP-positive multinuclear cells containing 3 or more nuclei were counted under the microscope as osteoclasts.

### 4.8. Flow Cytometry

Forty-eight hours after siRNA treatment, cells were detached from the culture dish and resuspended in FACS buffer. Then, the cells were incubated with anti-CD16/32 mAb (BioLegend, San Diego, CA, USA) for FcR blocking for 30 min and then stained with FITC-conjugated anti-Sema4D mAb (BioLegend, San Diego, CA, USA) and PE-conjugated anti-DC-STAMP mAb [43] for 30 more minutes. Cells were subjected to flow cytometry analysis using the BD FACSCanto™ II system (BD Biosciences, San Jose, CA, USA). Acquired data were analyzed using FlowJo v 10 software (BD Biosciences).

### 4.9. In Vitro Osteogenesis Assay

MC3T3-E1 osteoblast progenitor cells (ATCC, Manassas, VA, USA) were incubated i (1 × 10^5^ cells/well in a 48-well plate) in the culture medium supplemented with ascorbic acid (50 μg/mL) and β-glycerophosphate (5 mM) in the presence or absence of siRNA/C12-GD-Spe. After 7 days of incubation, MC3T3-E1 cells were subject to Alkaline Phosphatase (ALP) staining by reacting with a substrate solution containing NBT (nitro-blue tetrazolium chloride) and BCIP (5-bromo-4-chloro-3′-indolyphosphate p-toluidine salt) (Thermo Fisher Scientific).

### 4.10. Mouse Model of Ligature-Induced Periodontitis and siRNA Transfection 

Animal experiments were carried out in accordance with the institutional guidelines of Nova Southeastern University (IACUC approval #TK3). Periodontitis was induced in 6- to 8-week-old male C57BL/6 mice by placing silk ligatures (5-0; Ethicon) in a subgingival position around the cervix of maxillary second molar in each animal, as previously reported [24,43], while non-ligatured mice were counted as control. One week after ligature placement, a mixture of negative control siRNA or xOC-STAMP-siRNA (4 μg) included in C-12-GD-Spe, respectively, was injected below the gumline via 30 G needle. One week after the siRNA injection, mice were euthanized, and alveolar bone resorption was evaluated by μCT (see Section 4.9).

### 4.11. Micro-CT Imaging and Assessment of Alveolar Bone Resorption

The maxillary jawbones of euthanized mice were scanned by μCT (Skyscan 1176, Bruker), and alveolar bone resorption of each group was measured, as previously described [31]. In brief, total alveolar bone loss was calculated by measuring the distance from cemento-enamel junction (CEJ) to alveolar bone crest (ABC) from scanned images. The value obtained was divided by crown size of the same tooth, and the results of alveolar bone resorption were expressed in relative ratio.

### 4.12. Collection of the Local Tissue and Evaluation of mRNA Expression of the Tissue

Total RNA was extracted from periodontal tissues isolated from mice using PureLink™ RNA Mini Kit (Thermo Fisher Scientific) following the manufacturer’s protocol. The first strand cDNA was assembled from the extracted RNA (100 ng) using a Verso cDNA Synthesis Kit (Thermo Fisher Scientific). Amplification reactions were performed by Taqman Fast Advanced Master Mix (Thermo Fisher Scientific). After the activation of Taqman polymerase for 20 s at 95 °C. PCR was performed for a total of 40 cycles (1 s at 95 °C and 20 s at 60 °C) in the presence of specific primers (Thermo Fisher Scientific) for Gapdh, Alpl, and Acp5 in a QuantStudio™ 3 Q-PCR machine (Thermo Fisher Scientific). The ratios of specific gene’s mRNA level over that of control gene (Gapdh) were calculated using the ΔCt method (2^−ΔΔCt^) and expressed as “Relative mRNA expression”.

### 4.13. Statistical Analysis

Data are expressed as mean ± standard deviation (SD). Statistical significance was analyzed using Student’s *t*-test and ANOVA with Tukey–Kramer correction for multiple comparisons. *p* values < 0.05 were considered significant.

## 5. Conclusions

This study demonstrated that the complex of xOC-STAMP-siRNA and C12-GD-Spe could significantly downregulate RANKL-induced osteoclastogenesis in vitro and that local injection of xOC-STAMP-siRNA/C12-GD-Spe complex could attenuate bone resorption induced in a mouse model of periodontitis. These findings suggest that OC-STAMP is a promising target for the development of a novel bone regenerative therapy and that C12-GD-Spe gel provides a novel nanocarrier platform to deliver gene therapy for osteolytic diseases.

## Data Availability

The original contributions presented in the study are included in the article/Supplementary Materials; further inquiries can be directed to the corresponding author.

## Supplementary Materials

The following supporting information can be downloaded at https://www.mdpi.com/article/10.3390/gels10060377/s1. Table S1: Primer probes used for Quantitative Real-time RT-PCR in this study.
