# Supplementary material for: Cationic Glucan Dendrimer Gel-Mediated Local Delivery of Anti-OC-STAMP-siRNA for Treatment of Pathogenic Bone Resorption
Source: Gels. 2024 May 31;10(6):377. doi: 10.3390/gels10060377 (PMC11202495; doi:10.3390/gels10060377)
Supplement: Supplementary file 1 [file gels-10-00377-s001.zip › gels-2918012-supplementary.pdf]

Target	Supplier	Catalog number
Mouse GAPDH	Thermo Fisher Science	Mm99999915_g1
Mouse OC-STAMP	Thermo Fisher Science	Mm00512445_m1
Mouse DC-STAMP	Thermo Fisher Science	Mm04209236_m1
Mouse ACP5	Thermo Fisher Science	Mm00475698_m1
Mouse ALP	Thermo Fisher Science	Mm00475834_m1

Supporting Information Table S1

Primers used for real-time RT-PCR in this study.
